# Associations between serum vitamin D_3_, atherogenic indices of plasma and cardiometabolic biomarkers among patients with diabetes in the KERCADR study

**DOI:** 10.1186/s12902-022-01043-1

**Published:** 2022-05-12

**Authors:** Mohammad Reza Mahmoodi, Hamid Najafipour

**Affiliations:** 1 Physiology Research Center, Institute of Neuropharmacology, Kerman University of Medical Sciences, Kerman, Iran; 2 Department of Nutrition, Faculty of Public Health, Kerman University of Medical Sciences, Kerman, Iran. Haft Bagh-E-Alavi Highway, Kerman, 7616913555 Iran; 3 Department of Physiology and Pharmacology, Afzalipour Faculty of Medicine, Kerman University of Medical Sciences, Kerman, Iran

**Keywords:** 25-hydroxyvitamin D3, Atherogenic indices of plasma, Biomarkers of cardiometabolic risk, Type 2 diabetes

## Abstract

**Background:**

We sought the association between serum 25-hydroxyvitamin D_3_ (25(OH) D_3_) levels and atherogenic indices of plasma as novel predictive biomarkers of cardiometabolic disease risk in patients with type 2 diabetes mellitus (T2DM).

**Methods:**

The present study was a nested case-control study conducted on 252 participants with T2DM and controls from the second phase of the KERCADR cohort study. The participants with a mean (±SD) age of 49.79 ± 5.85 years were randomly selected and allocated into case and control groups. Independent t-test, Hierarchical Linear Regression, Univariate ANOVA, and partial correlation were used for analysis the data. Atherogenic indices of plasma include Castelli Risk Index I (CRI I), Castelli Risk Index II (CRI II), and the novel Atherogenic Index of Plasma (AIP), and Atherogenic Coefficient (AC).

**Results:**

There was a significant difference among case and control groups for AIP in males and females (*P* < 0.001 and *P* = 0.007, respectively). The levels of AIP, CRI I, and AC significantly decreased (*P* = 0.017, *P* = 0.029, and *P* = 0.029, respectively) with improved serum vitamin D status only in control male participants. The main effect of BMI and vitamin D status on AIP, CRI I, and AC, and the main effect of BMI on CRI I, CRI II, and AC were significant in control males and females, respectively.

**Conclusion:**

We conclude that there is a reverse significant association between AIP and serum vitamin D among healthy males. Low serum level of vitamin D is associated with atherogenic dyslipidemia. Therefore, improving vitamin D status as an important indicator may alleviate AIP as a surrogate marker for predicting the risk of CVD events in healthy men and women with normal BMI.

## Introduction

Low serum 25-hydroxyvitamin D_3_ [25(OH) D_3_] levels are significantly associated with a higher risk of developing prediabetes and type 2 diabetes mellitus (T2DM) in individuals [1, 2]. Epidemiologic studies reveal an association between low serum 25(OH) D_3_ level and an increased risk of metabolic syndrome (MS) and T2DM [3]. In addition, observational studies have shown that vitamin D adequacy can reduce the severity of T2DM, insulin resistance, prediabetes, and MS. However, there is a lack of convincing evidence from randomized control clinical trials that these complications are prevented following optimization of serum levels of 25(OH) D_3_ [4]. Although, the prevalence of hypovitaminosis D do not differ significantly in healthy adults; the mean of 25(OH) D_3_ decrease with an increasing number of cardiometabolic risk factors such as central obesity, hypertension, increased atherogenic risk, and insulin resistance [5]. The prevalence of low serum 25(OH) D_3_ levels is considerably high in patients with cardiovascular disease (CVD) risk factors. These patients present significantly higher values for cardiometabolic biomarkers such as fasting plasma glucose (FPG), hemoglobin A1c (HbA1c), total cholesterol (TC), triglyceride (TG), body mass index (BMI), waist circumference (WC), and atherogenic indices (Castelli Risk Index I (CRI I), Castelli Risk Index II (CRI II), and Atherogenic index of plasma (AIP)) [6]. Serum vitamin D correlated negatively with glycemia, HbA1C, TG, atherogenic indices, BMI, and hypertension [6]. There is a significant negative correlation of serum vitamin D with lipid markers and atherogenic variables in poor glycemic control diabetic patients. The serum vitamin D levels were inversely associated with HbA_1_c, FPG, TG, TC, and non-HDL-C [7].

There is a positive and significant correlation between AIP and cardiometabolic risk factors such as BMI, TG, WC, TC, low-density lipoprotein cholesterol (LDL-C), HbA1c, FPG, systolic blood pressure (SBP), diastolic blood pressure (DBP) in many studies [8–10]. Therefore, AIP can be used as a surrogate marker both for predicting the risk of CVD risk factors and, additionally, it has been shown that AIP is associated with subclinical atherosclerosis and CVD events in both healthy women and CVD postmenopausal women [8–11].

Vitamin D deficiency as a modifiable risk factor is associated with a worse cardiometabolic risk profile. A positive and significant association between AIP and higher HbA1c, CRI I, and lower HDL-C are seen in people with plasma 25(OH) D_3_ less than 25 nmol/L [12]. Serum levels of HDL-C, 25(OH) D_3_, free vitamin D and bioavailable vitamin D are significantly lower in diabetic patients than in non-diabetic patients while TG and remnant cholesterol levels are found to be significantly higher [13]. In a study, the majority of Korean adults with prediabetes have a serum 25(OH) D_3_ less than 20 ng/ml, and the proportion of adults having low HDL-C is the highest among the vitamin D deficiency group [14]. Partial correlations adjusting for age and sex show that vitamin D concentrations are significantly inversely associated with AIP and visceral adiposity index in both males and females [15]. In another study, the serum 25(OH) D levels are closely associated with the serum lipids and AIP. Vitamin D deficiency is associated with an increased risk of dyslipidemias, especially in men. Accordingly, the association between vitamin D status and AIP varies by gender [16]. 25(OH) D and AIP are significantly different between control and T2DM groups. Serum 25(OH) D showed a significant negative correlation with AIP among total study subjects. The association between 25(OH) D and various CVD risk markers suggests that 25(OH) D might help in the prediction of CVD risk [17]. A progressive decrease in TC, LDL-C, and non-HDL-C is revealed as the serum vitamin D level increased. There is a negative linear association between 25(OH) D and TC, LDL-C, and non-HDL-C in obese patients [18].

Considering the limited studies regarding the association between serum 25(OH) D_3_ levels and atherogenic indices of plasma in patients with DM, this study aims to assess vitamin D status in participants and to find out whether there is an association between serum 25(OH) D_3_ levels and atherogenic indices of plasma as novel surrogate markers as well as biomarkers of cardiometabolic disease risk in patients with DM and a healthy population based on genders in KERCADR study as an Iranian community.

## Materials and methods

### Participants eligibility and study design

The present study is a nested case-control study conducted on participants with type 2 diabetes and controls from the second phase of the KERCADR cohort study. For each case, a healthy matched control was selected from among participants in the KERCADR cohort study. The second phase of KERCADR is a cohort study on over 10,000 individuals aged 15-75 years old who were recruited in the household survey on Kerman province residences. The baseline protocol, the sampling method, and the recruitment have been previously described in detail [19,20]. Kerman province is one of the 31 provinces of Iran. Kerman is in the southeast of Iran with its administrative center in the city of Kerman.

Two hundred fifty-two participants (136 males and 116 females) were randomly selected and the total number of participants with diabetes was 124 (69 males and 55 females) and controls was 128 (67 males and 61 females) from the KERCADR study. The management of confounding variables in study design and to ensure that the study groups did not differ concerning effective confounders as inclusion criteria, the cases to controls ratio became 1:1. Limiting the study to participants in effective confounders was a simple technique of ensuring that all participants have the same level of the confounder.

The criteria for eligibility of participants with diabetes mellitus were 1) willingness to participate in the study and sign the informed consent, 2) the presence of type 2 diabetes at least for one year 3) patients receive either diet therapy or diet therapy with a combination of oral anti-diabetic medications, 4) no history of myocardial infarction, stroke, cardiovascular disease, active cancer, liver, kidney, and thyroid dysfunction, and infectious diseases, 5) no history of high blood pressure, 6) BMI lower than 30 and from both genders. The criteria for eligibility of control participants were 1) willingness to participate in the study and sign the informed consent, 2) no history of diabetes mellitus, 3) no history of myocardial infarction, stroke, cardiovascular disease, active cancer, liver, kidney, and thyroid dysfunction, and infectious diseases, 4) no history of high blood pressure, 5) BMI lower than 30 and from both of genders that matched with case participants.

The existence of any of the exclusion criteria among participants may profoundly affect plasma atherogenic indices and the other cardiovascular biomarkers. Therefore, compliance with all of these criteria would result in greater transparency of the association between vitamin D levels and plasma atherogenic indices.

The protocol was approved by review panels and ethics committees (Approval ID: IR.KMU.REC. 1399.405) of the Vice-chancellor for Research of Kerman University of Medical Sciences.

### Clinical and biochemical examinations

Blood samples were drawn into EDTA tubes after a 12-14 h fast at the study baseline. Plasma samples were stored at − 80 °C until a final assay for glycemic and lipoprotein biomarkers could be performed. Cardiometabolic biomarkers include FBS, HbA1c, TC, HDL-C, LDL-C, TG, SBP, DBP, WC, hip circumference (HC), waist to hip ratio (WHR), weight, and BMI.

As previously described in the other studies [19, 20], all measurements were performed according to the standard protocol. The patients fasted for 12-14 h before admission. FBS (KIMIA Kit, Code 890410, Iran) was measured using the glucose oxidase-peroxidase method. HDL-C (PARS Kit, Code 89022, Iran) and TG (KIMIA Kit, Code 890201, Iran), were measured by standard enzymatic procedures. BP was recorded using an automated oscillometric BP monitor (standard mercury manometer–Model RIESTER, Germany) after at least 10 min of rest in a chair and arm supported at heart level. TC (KIMIA Kit, Code 890303, Iran) and LDL-C were calculated based on the Friedewald formula [LDL-C = TC – (HDL-C + TG/5)]. HbA1C (NYCOCARD Kit, Code 1042184, Austria) was determined based on Bio-rad Variant High-Performance Liquid Chromatography [HPLC] assay.

### Atherogenic indices of plasma

Atherogenic indices of plasma include Castelli Risk Index I, Castelli Risk Index II, and the novel Atherogenic Index of Plasma, and Atherogenic Coefficient. AIP or TG/high-density lipoprotein cholesterol (TG/HDL-C) ratio is a logarithmic transformation of the ratio of molar concentrations of TG to HDL-C. CRI I is the ratio of TC to HDL-C, and CRI II is the ratio of LDL-C to HDL-C. AC is the ratio of non-HDL-C to HDL-C. These parameters are being applied for assessing cardiovascular risk.

### Determination of 25-hydroxyvitamin D_3_

Blood samples were drawn into tubes after a 12-14 h fast and immediately stored at − 80 °C until an assay for 25-hydroxyvitamin D_3_ could be performed. Serum 25-hydroxyvitamin D_3_ was measured by an enzyme-linked immune sorbent assay (ELISA) (Monobind 25-OH Vit D [Direct]) with the use of an automated analyzer with a sensitivity of ng/ml through the protocol of the ELISA kit.

### Anthropometry assessment

Weight and BMI (the weight in kilograms divided by the square of the height in meters) were measured and recorded in questionnaires. WC was measured at the umbilical level using a non-stretchable measuring tape, without any pressure to the body surface.

### Statistical analysis

Statistical analysis was performed using IBM SPSS Statistics software, version 22.0. The normal distribution of biomarkers was investigated by the Kolmogorov-Smirnov test. Significance was assumed at *P* < 0.05. The mean differences of the cardiometabolic biomarkers, the levels of atherogenic indices of plasma, and serum vitamin D_3_ between the case and the control participants in each gender group were compared by independent t-test (Tables 1 & 2). The one-way analysis of variance (ANOVA) in which all pairwise comparisons among the three vitamin D statuses (vitamin D deficiency with serum vitamin D ≤ 20 ng/ml, vitamin D insufficiency with serum vitamin D > 20-30 ng/ml, and vitamin D satisfactory with serum vitamin D > 30 ng/ml) were performed with the use of Tukey’s HSD honestly significant difference procedure (Table 3). Hierarchical Linear Regression was applied to investigate whether adding atherogenic indices of plasma as independent variables (predictors) significantly improves a model’s ability to predict serum 25(OH) vitamin D3 as a dependent variable and/or to investigate a moderating effect of a variable (Table 4).

**Table 1:** Mean ± SE of Cardiometabolic Biomarkers of Participants in Case Control Study according to Gender

	Total Participants (*n =* 252)		*P value*	Male (*n =* 136)		*P value*	Female (*n =* 116)		*P value*
	Case (*n =* 124)	Control (*n =* 128)		Case (*n =* 69)	Control (*n =* 67)		Case (*n =* 55)	Control (*n =* 61)	
Age (Year)	51.3 ± 0.5	50.1 ± 0.5	0.086	49.0 ± 0.6	49.8 ± 0.7	0.355	45.7 ± 0.7	46.0 ± 0.7	0.726
Weight (Kg)	70.6 ± 1.0	68.1 ± 0.9	0.061	74.3 ± 1.2	71.4 ± 1.3	0.105	65.9 ± 1.2	64.5 ± 1.2	0.411
Height (m)	164.2 ± 0.8	164.7 ± 0.8	0.689	169.6 ± 0.8	170.9 ± 0.8	0.257	157.4 ± 0.8	157.8 ± 0.7	0.677
Body Mass Index (Kg/m^2^)	26.1 ± 0.3	25.1 ± 0.3	**0.013**	25.8 ± 0.3	24.5 ± 0.4	**0.014**	26.5 ± 0.4	25.9 ± 0.4	0.250
Waist Circum. (cm)	91.4 ± 0.8	88.7 ± 0.9	**0.027**	93.9 ± 0.9	90.4 ± 1.1	**0.015**	88.2 ± 1.3	86.8 ± 1.4	0.480
Hip Circum. (cm)	98.8 ± 0.63	99.1 ± 0.7	0.708	99.2 ± 0.8	98.5 ± 0.9	0.559	98.2 ± 1.0	99.8 ± 0.9	0.245
Waist to Hip ratio	0.93 ± 0.01	0.89 ± 0.01	**0.000**	0.95 ± 0.01	0.92 ± 0.01	**0.000**	0.90 ± 0.01	0.87 ± 0.01	**0.039**
Fasting Blood Sugar (mg/dl)	181.3 ± 6.1	90.1 ± 1.0	**0.000**	177.5 ± 8.5	90.4 ± 1.2	**0.000**	186.1 ± 8.8	89.7 ± 1.5	**0.000**
HbA1c	8.4 ± 0.3	–	–	8.5 ± 0.3	–	–	8.2 ± 0.4	–	–
Serum triglyceride (mg/dl)	184.7 ± 9.9	120.7 ± 5.6	**0.000**	181.9 ± 13.9	127.8 ± 8.0	**0.001**	188.3 ± 14.1	112.9 ± 7.8	**0.000**
Total Cholesterol (mg/dl)	193.1 ± 3.9	183.0 ± 3.0	**0.043**	191.7 ± 5.1	182.7 ± 4.5	0.191	194.8 ± 6.2	183.3 ± 4.0	0.120
LDL-C (mg/dl)	111.8 ± 3.7	114.4 ± 2.5	0.549	111.8 ± 5.0	114.0 ± 3.4	0.720	111.7 ± 5.4	114.9 ± 3.8	0.619
HDL-C (mg/dl)	45.2 ± 0.9	44.4 ± 0.9	0.562	44.5 ± 1.0	43.1 ± 1.3	0.411	46.1 ± 1.6	45.9 ± 1.3	0.922
Systolic blood pressure (mmHg)	112.7 ± 1.1	110.4 ± 1.1	0.151	113.1 ± 1.5	113.4 ± 1.4	0.971	112.0 ± 1.6	107.1 ± 1.8	**0.044**
Diastolic blood pressure (mmHg)	74.0 ± 0.8	71.7 ± 0.9	0.056	74.0 ± 1.03	73.8 ± 1.2	0.887	73.9 ± 1.1	69.3 ± 1.4	**0.013**

**Table 2:** Mean ± SE and Interquartile Range^a^ of Serum 25(OH) Vit. D and Atherogenic Indices^b^ of Plasma of Participants according to Gender

	Total Participants (*n =* 252)		*P value*	Male (*n =* 136)		*P value*	Female (*n =* 116)		*P value*
	Case	Control		Case (*n =* 69)	Control (*n =* 67)		Case (*n =* 55)	Control (i61)	
Serum 25(OH)D^c^	26.6 ± 1.1 (18.0)	25.8 ± 1.1 (17.75)	0.656	26.4 ± 1.3 (15.0)	26.1 ± 1.2 (13.0)	0.677	26.8 ± 2.1 (23.0)	25.6 ± 1.9 (23.0)	0.860
AIP	0.56 ± 0.03 (0.41)	0.39 ± 0.02 (0.35)	**0.000**	0.56 ± 0.03 (0.35)	0.43 ± 0.03 (0.37)	**0.000**	0.57 ± 0.04 (0.49)	0.35 ± 0.03 (0.34)	**0.007**
CRI I	4.45 ± 0.12 (1.58)	4.26 ± 0.08 (1.33)	0.200	4.44 ± 0.14 (1.75)	4.37 ± 0.12 (1.17)	0.174	4.47 ± 0.21 (1.60)	4.14 ± 0.12 (1.55)	0.706
CRI II	2.50 ± 0.09 (1.09)	2.68 ± 0.07 (1.17)	0.109	2.49 ± 0.10 (1.14)	2.74 ± 0.09 (0.93)	0.557	2.50 ± 0.15 (1.18)	2.61 ± 0.10 (1.27)	0.079
AC	3.45 ± 0.12 (1.58)	3.26 ± 0.08 (1.33)	0.200	3.44 ± 0.14 (1.75)	3.37 ± 0.12 (1.17)	0.174	3.47 ± 1.59 (1.60)	3.14 ± 0.12 (1.55)	0.706

^a^ Interquartile range is in bracket

^b^ Atherogenic Index of Plasma = AIP; Castelli Risk Index I=CRI I; Castelli Risk Index II=CRI II; Atherogenic Coefficient = AC

^c^ Based on ng/ml

**Table 3:** Mean ± SE^a^ of Atherogenic Indices^b^ of Plasma according to Vitamin D Status^c^ of Participants

Atherogenic Indices	Vitamin D Status (Male)			*P value*	Vitamin D Status (Female)			*P value*
	Vitamin D Deficiency	Vitamin D Insufficiency	Vitamin D Satisfactory		Vitamin D Deficiency	Vitamin D Insufficiency	Vitamin D Satisfactory	
Case group	(*n =* 19)	(*n =* 29)	(*n =* 21)		(*n =* 23)	(i10)	(i22)	
AIP	0.62 ± 0.06	0.52 ± 0.05	0.55 ± 0.06	0.523	0.60 ± 0.06	0.44 ± 0.09	0.59 ± 0.07	0.310
CRI I	4.71 ± 0.20	4.34 ± 0.23	4.37 ± 0.32	0.556	4.31 ± 0.27	4.04 ± 0.26	4.84 ± 0.43	0.351
CRI II	2.72 ± 0.16	2.41 ± 0.16	2.43 ± 0.22	0.447	2.28 ± 0.17	2.38 ± 0.16	2.82 ± 0.34	0.275
AC	3.71 ± 0.20	3.34 ± 0.23	3.37 ± 0.32	0.556	3.31 ± 0.27	3.04 ± 0.26	3.84 ± 0.43	0.351
Control group	(i21)	(*n =* 23)	(*n =* 23)		(*n =* 27)	(*n =* 13)	(*n =* 21)	
AIP	0.55 ± 0.06^d^	0.39 ± 0.05	0.36 ± 0.04	**0.017**	0.35 ± 0.06	0.34 ± 0.06	0.36 ± 0.04	0.977
CRI I	4.76 ± 0.25^d^	4.37 ± 0.19	4.01 ± 0.13	**0.029**	4.10 ± 0.19	4.11 ± 0.28	4.26 ± 0.21	0.792
CRI II	2.93 ± 0.21	2.79 ± 0.15	2.50 ± 0.11	0.166	2.49 ± 0.13	2.62 ± 0.25	2.75 ± 0.20	0.553
AC	3.76 ± 0.25^d^	3.37 ± 0.19	3.01 ± 0.13	**0.029**	3.10 ± 0.19	3.11 ± 0.28	3.26 ± 0.21	0.792

^a^ One-way ANOVA analyzed the differences (M ± SE) atherogenic indices between three vitamin D statuses for each gender group in case and control groups

^b^ Atherogenic Index of Plasma = AIP; Castelli Risk Index I=CRI I; Castelli Risk Index II=CRI II; Atherogenic Coefficient = AC

c Vitamin D status divided into vitamin D deficiency (serum vitamin D ≤ 20 ng/ml), vitamin D insufficiency (serum vitamin D > 20-30 ng/ml), and vitamin D satisfactory (serum vitamin D > 30 ng/ml)

^d^ There are statistically significant differences between vitamin D deficiency and vitamin D satisfactory

**Table 4:** Hierarchical Linear Regression for Serum Vitamin D^a^ and Atherogenic Indices of Plasma of Case and Control Participants

	Unstandardized Coefficients		Standardized Coefficients	t	Sig.	95.0% Confidence Interval for B	
Model^b^/Case	B	Std. Error	Beta			Lower Bound	Upper Bound
1 (Constant)	27.496	2.580		10.656	0.000	22.388	32.604
AIP	−1.685	4.085	−0.037	− 0.412	0.681	−9.773	6.402
2 (Constant)	24.013	4.010		5.988	0.000	16.073	31.953
AIP	−6.338	5.787	−0.140	−1.095	0.276	−17.796	5.120
CRI I	1.367	1.206	0.145	1.134	0.259	−1.020	3.755
3 (Constant)	24.216	4.198		5.769	0.000	15.900	32.531
AIP	−10.293	8.514	−0.212	−1.209	0.229	−27.159	6.574
CRI I	2.073	3.098	0.213	0.669	0.505	−4.065	8.210
CRI II	−0.595	3.241	−0.047	− 0.184	0.855	−7.016	5.825
4 (Constant)	28.026	4.815		5.821	0.000	18.487	37.564
AIP	−12.551	8.578	−0.258	−1.463	0.146	−29.545	4.444
CRI I	3.092	3.145	0.317	0.983	0.328	−3.138	9.322
CRI II	0.713	3.324	0.056	0.214	0.831	−5.873	7.299
Non-HDL	−0.071	0.045	−0.233	−1.583	0.116	−0.159	0.018
5 (Constant)	31.118	4.823		6.452	0.000	21.562	40.673
AIP	−12.551	8.578	−0.258	−1.463	0.146	−29.545	4.444
CRI II	0.713	3.324	0.056	0.214	0.813	−5.873	7.299
Non-HDL	−0.071	0.045	−0.233	−1.583	0.116	−0.159	0.018
AC	3.092	3.145	0.317	0.983	0.328	−3.138	9.322
Model/Control							
1 (Constant)	28.336	2.105		13.464	0.000	24.171	32.500
AIP	−6.353	4.553	−0.123	−1.395	0.165	−15.362	2.657
2 (Constant)	29.641	5.465		5.424	0.000	18.826	40.547
AIP	−5.275	6.180	−0.102	−0.857	0.395	−17.505	6.956
CRI I	−0.406	1.567	−0.031	−0.259	0.796	−3.508	2.696
3 (Constant)	47.017	10.005		4.699	0.000	27.214	66.820
AIP	20.133	13.749	0.391	1.464	0.146	−7.080	47.346
CRI I	−17.627	8.493	−1.350	−2.075	**0.040**	−34.438	−0.816
CRI II	17.184	8.333	1.067	2.062	**0.041**	0.690	33.678
4 (Constant)	48.017	10.269		4.676	0.000	27.690	68.344
AIP	19,877	13.804	0.386	1.440	0.152	−7.447	47.201
CRI I	−17.250	8.560	−1.321	−2.015	**0.046**	−34.193	−0.307
CRI II	17.405	8.374	1.081	2.079	**0.040**	0.830	33.980
Non-HDL	−0.022	0.048	−0.056	− 0.461	0.646	− 0.118	0.074
5 (Constant)	30.767	5.013		6.137	0.000	20.843	40.690
AIP	19.877	13.804	0.386	1.440	0.152	−7.447	47.201
CRI II	17.405	8.374	1.081	2.079	**0.040**	0.830	33.980
Non-HDL	−0.022	0.048	−0.056	− 0.461	0.646	− 0.118	0.074
AC	−17.250	8.560	−1.321	−2.051	**0.046**	−34.193	−0.307

^a^ Dependent variable: Serum 25(OH) vitamin D3

^b^ Model 1. Predictors: (constant), Atherogenic Index of Plasma

Model 2. Predictors: (constant), Castelli Risk Index I, Atherogenic Index of Plasma

Model 3. Predictors: (constant), Castelli Risk Index II, Atherogenic Index of Plasma, Castelli Risk Index I

Model 4. Predictors: (constant), Non-HDL-C, Atherogenic Index of Plasma, Castelli Risk Index II, Castelli Risk Index I

Model 5. Predictors: (constant), Atherogenic Coefficient, Atherogenic Index of Plasma, Non-HDL-C, Castelli Risk Index II

Then, general linear models (Univariate ANOVA) were used to find out whether the interaction between two independent variables such as vitamin D concentration and BMI on atherogenic indices of plasma is significant. As previously described, serum vitamin D status divided into deficiency, insufficiency, and satisfactory statuses. BMI dichotomized into BMI equal or less than 26.00 and greater than 26.00 in the case males and control females and dichotomized into BMI equal or less than 26.50 and greater than 26.50 in the case females; and dichotomized into BMI equal or less than 25.00 and greater than 25.00 in the control males (Table 5). Because the mean and median of the independent variable BMI (less than or equal and greater than median BMI) have closely corresponded, this variable was dichotomized. The assumptions for ANOVA and Univariate ANOVA principally the assumption of homogeneity of variances have been met.

**Table 5:** Univariate Analysis of Variances^a^ of Two Independent Vitamin D Statuses^b^ and Body Mass Index^c^ on Atherogenic Indices of Plasma

	Vitamin D Deficiency		Vitamin D Insufficiency		Vitamin D Satisfactory		Sig. Vitamin D	Sig. BMI	Sig. Vit D^a^BMI
	≤ median BMI	> median BMI	≤ median BMI	> median BMI	≤ median BMI	> median BMI			
*Male*									
Atherogenic Index of Plasma									
Case	0.54 ± 0.11	0.66 ± 0.08	0.46 ± 0.08	0.56 ± 0.07	0.55 ± 0.08	0.56 ± 0.11	0.596	0.290	0.846
Control	0.45 ± 0.07	0.63 ± 0.06	0.29 ± 0.05	0.59 ± 0.07	0.35 ± 0.06	0.38 ± 0.06	**0.025**	**0.002**	0.112
Castelli Risk Index I									
Case	4.40 ± 0.46	4.90 ± 0.35	4.12 ± 0.35	4.50 ± 0.30	4.58 ± 0.33	3.95 ± 0.46	0.583	0.787	0.288
Control	4.34 ± 0.29	5.08 ± 0.25	4.10 ± 0.23	4.88 ± 0.31	3.85 ± 0.26	4.16 ± 0.25	**0.029**	**0.007**	0.622
Castelli Risk Index II									
Case	2.61 ± 0.32	2.79 ± 0.26	2.44 ± 0.25	2.39 ± 0.21	2.58 ± 0.24	2.16 ± 0.32	0.461	0.665	0.584
Control	2.67 ± 0.24	3.13 ± 0.21	2.67 ± 0.19	3.01 ± 0.26	2.36 ± 0.22	2.65 ± 0.21	0.165	0.052	0.923
Atherogenic Coefficient									
Case	3.40 ± 0.46	3.90 ± 0.35	3.12 ± 0.35	3.50 ± 0.30	3.58 ± 0.33	2.95 ± 0.46	0.583	0.787	0.288
Control	3.34 ± 0.29	4.10 ± 0.25	3.10 ± 0.23	3.87 ± 0.31	2.85 ± 0.26	3.16 ± 0.25	**0.029**	**0.007**	0.622
*Female*									
Atherogenic Index of Plasma									
Case	0.58 ± 0.09	0.62 ± 0.09	0.49 ± 0.18	0.42 ± 0.12	0.61 ± 0.10	0.58 ± 0.09	0.441	0.834	0.873
Control	0.30 ± 0.07	0.40 ± 0.07	0.29 ± 0.12	0.36 ± 0.08	0.27 ± 0.09	0.40 ± 0.07	0.948	0.138	0.939
Castelli Risk Index I									
Case	4.28 ± 0.47	4.35 ± 0.49	4.05 ± 0.94	4.04 ± 0.61	4.60 ± 0.51	5.04 ± 0.47	0.416	0.735	0.908
Control	3.68 ± 0.26	4.43 ± 0.25	3.60 ± 0.47	4.34 ± 0.32	3.95 ± 0.36	4.41 ± 0.25	0.823	**0.019**	0.868
Castelli Risk Index II									
Case	2.14 ± 0.34	2.41 ± 0.34	2.24 ± 0.65	2.44 ± 0.43	2.52 ± 0.38	3.06 ± 0.34	0.317	0.340	0.908
Control	2.24 ± 0.22	2.75 ± 0.21	2.19 ± 0.40	2.81 ± 0.27	2.58 ± 0.30	2.84 ± 0.21	0.625	**0.043**	0.800
Atherogenic Coefficient									
Case	3.28 ± 0.47	3.35 ± 0.49	3.05 ± 0.94	3.04 ± 0.61	3.60 ± 0.51	4.04 ± 0.47	0.416	0.735	0.908
Control	2.68 ± 0.26	3.43 ± 0.25	2.60 ± 0.47	3.34 ± 0.32	2.95 ± 0.36	3.41 ± 0.25	0.823	**0.019**	0.868

^a^ Univariate Analysis of Variances analyzed the differences (Mean ± SE) atherogenic indices between serum vitamin D concentrations based on three vitamin D statuses and body mass index based on median for each gender group in case and control groups

^b^ Vitamin D status divided into vitamin D deficiency (serum vitamin D ≤ 20 ng/ml), vitamin D insufficiency (serum vitamin D > 20-30 ng/ml), and vitamin D satisfactory (serum vitamin D > 30 ng/ml)

^c^ Body mass index dichotomized into body mass index ≤25.99 and > 26.00 in male case group and dichotomized into body mass index ≤26.49 and > 26.50 in female case group; and dichotomized into body mass index ≤24.99 and > 25.00 in male control group and dichotomized into body mass index ≤25.99 and > 26.00 in female control group

The Pearson correlation coefficient was calculated to examine the relationship between atherogenic indices as well as between serum vitamin D and lipid and lipoprotein profiles (Tables 6 & 7).

**Table 6:** Pearson Correlation Coefficients for the Relationship between Atherogenic Indices of Plasma according to gender

Atherogenic Indices	Atherogenic Index of Plasma	Castelli Risk Index I	Castelli Risk Index II	Atherogenic Coefficient	Atherogenic Index of Plasma	Castelli Risk Index I	Castelli Risk Index II	Atherogenic Coefficient
Case	**Male**				**Female**			
Atherogenic Index of Plasma	1	0.701 **< 0.001**	−0.003 0.979	0.701 **< 0.001**	1	0.727 **< 0.001**	0.440 **< 0.001**	0.727 **< 0.001**
Castelli Risk Index I		1	0.686 **< 0.001**	1.000 **< 0.001**		1	0.957 **< 0.001**	1.000 **< 0.001**
Castelli Risk Index II			1	0.686 **< 0.001**			1	0.957 **< 0.001**
Atherogenic Coefficient				1				1
Control	**Male**				**Female**			
Atherogenic Index of Plasma	1	0.717 **< 0.001**	0.429 **< 0.001**	0.717 **< 0.001**	1	0.615 **< 0.001**	0.294 **< 0.001**	0.615 **< 0.001**
Castelli Risk Index I		1	0.929 **< 0.001**	1.000 **< 0.001**		1	0.920 **< 0.001**	1.000 **< 0.001**
Castelli Risk Index II			1	0.929 **< 0.001**			1	0.920 **< 0.001**
Atherogenic Coefficient				1				1

**Table 7:** Pearson Correlation Coefficients for the Relationship between Serum Vitamin D and the Lipid and Lipoprotein Profiles according to gender

Biomarkers	Serum Vitamin D	Triglyceride	Total Cholesterol	LDL-Chol	HDL-Chol	Serum Vitamin D	Triglyceride	Total Cholesterol	LDL-Chol	HDL-Chol
Case	**Male**					**Female**				
Serum Vitamin D	1	−0.147 0.237	−0.280 **0.020**	− 0.204 0.101	0.053 0.671	1	−0.070 0.628	0.148 0.301	0.217 0.125	−0.057 0.694
Triglyceride		1	0.126 0.313	−0.270 **0.029**	−0.430 **< 0.001**		1	0.260 0.065	0.042 0.772	−0.401 **0.004**
Total Cholesterol			1	0.901 **< 0.001**	0.346 **0.004**			1	0.948 **< 0.001**	0.394 **< 0.001**
LDL-Chol				1	0.389 **0.001**				1	0.306 **0.029**
HDL-Chol					1					1
Control		**Male**				**Female**				
Serum Vitamin D	1	−0.238 0.053	−0.152 0.221	−0.134 0.281	0.110 0.376	1	−0.088 0.500	0.015 0.907	0.059 0.654	−0.017 0.896
Triglyceride		1	0.448 **< 0.001**	0.164 0.184	−0.097 0.435		1	0.198 0.127	−0.126 0.332	−0.237 0.066
Total Cholesterol			1	0.914 **< 0.001**	0.532 **< 0.001**			1	0.896 **< 0.001**	0.217 0.093
LDL-Chol				1	0.363 0.003				1	−0.016 0.904
HDL-Chol					1					1

## Results

### Characteristics of participants

The mean (±SD) age of participants was 49.79 ± 5.85 years (cases, 47.54 ± 5.49; controls, 48.05 ± 5.80). Fifty-four percent of the participants were male.

### Cardiometabolic biomarkers of participants

The Cardiometabolic biomarkers of participants in case and control groups according to gender are shown in Table 1. There were significant differences among case and control males for BMI, WC, WHR, FBS, and TG (*P* = 0.014, *P* = 0.015, *P* < 0.001, *P* < 0.001, and *P* = 0.001, respectively) and among females for WHR, FBS, TG, SBP, and DBP (*P* = 0.039, *P* < 0.001, *P* < 0.001, *P* = 0.044 and *P* = 0.013, respectively) (Table 1).

### Serum 25(OH) D_3_ and atherogenic indices of participants

Table 2 indicates the mean (±SE) and interquartile range of serum 25(OH) D_3_ and atherogenic indices of plasma of participants according to gender. There was a significant difference among case and control groups for AIP in males and females (*P* < 0.001 and *P* = 0.007, respectively). There were no significant differences in serum 25(OH) D_3_ and the other atherogenic indices of plasma of participants among the case and control groups according to gender (Table 2).

### Atherogenic indices according to vitamin D status of participants

Table 3 indicates the mean (±SE) of atherogenic indices of plasma according to the vitamin D status of participants. Results of one-way ANOVA showed that the levels of AIP, CRI I, and AC significantly decreased (*P* = 0.017, *P* = 0.029, and *P* = 0.029, respectively) with improved serum vitamin D status only in control male participants. The levels of atherogenic indices of plasma non-significantly decreased with improved serum vitamin D status only in case male participants.

### Results from hierarchical linear regression

To investigate whether adding atherogenic indices of plasma as independent variables significantly improve a model’s ability to predict serum 25(OH) vitamin D3 as a dependent variable, Hierarchical Linear Regression was applied. Table 4 quantifies the relationship between predictor variables and serum vitamin D. Results identifies none of the atherogenic indices of plasma impact on serum vitamin D of case participants. However, results showed that CRI I and CRI II in models 3 and 4 (*P* = 0.040 and 0.041; and 0.046 and 0.040 respectively) as well as CRI II and AC in model 5 (*P* = 0.040 and 0.046) have a slight impact on serum vitamin D of control participants. It seems that models 3 and 4 are the right models to determine variables associated with serum vitamin D as a dependent variable for control participants (Table 4). However, due to the number of participants studied in each gender, the gender effect was not investigated in the model, separately. Therefore, caution should be taken to consider the analysis of the genders in the models.

### Interaction between BMI and serum 25(OH) Vit. D on atherogenic indices

To find out whether the interaction between two independent variables such as three vitamin D statuses and BMI on atherogenic indices of plasma is significant, Univariate ANOVA was applied. The Univariate ANOVA indicated interaction results between two independent variables on atherogenic indices of participants. The interaction between BMI and vitamin D status on atherogenic indices were not significant in case and control participants. However, the main effect of BMI and vitamin D status on AIP, CRI I, and AC were significant in control males. The main effect of BMI on CRI I, CRI II, and AC were significant in control females.

### Results from bivariate and partial correlation

Table 6 indicates the Pearson correlation coefficient between atherogenic indices. The strong significant relationships were found between AIP with CRI I and AC for males (0.701-0.717) and females (0.615-0.727) in both case and controls (*P* < 0.001). However, very strong significant relationships were found between CRI I with CRI II (0.920-0.957), CRI II with AC (0.920-0.957), and CRI I with AC (1.000) for control males and females and case females (*P* < 0.001) (Table 6).

A weak significant negative relationship was found between serum vitamin D with TC for case males (*r* = − 0.280, *P* < 0.020). However, the partial correlation adjusting for BMI and WHR did not change the significance of the relationship between the biomarkers (Table 7).

**Fig. 1:**
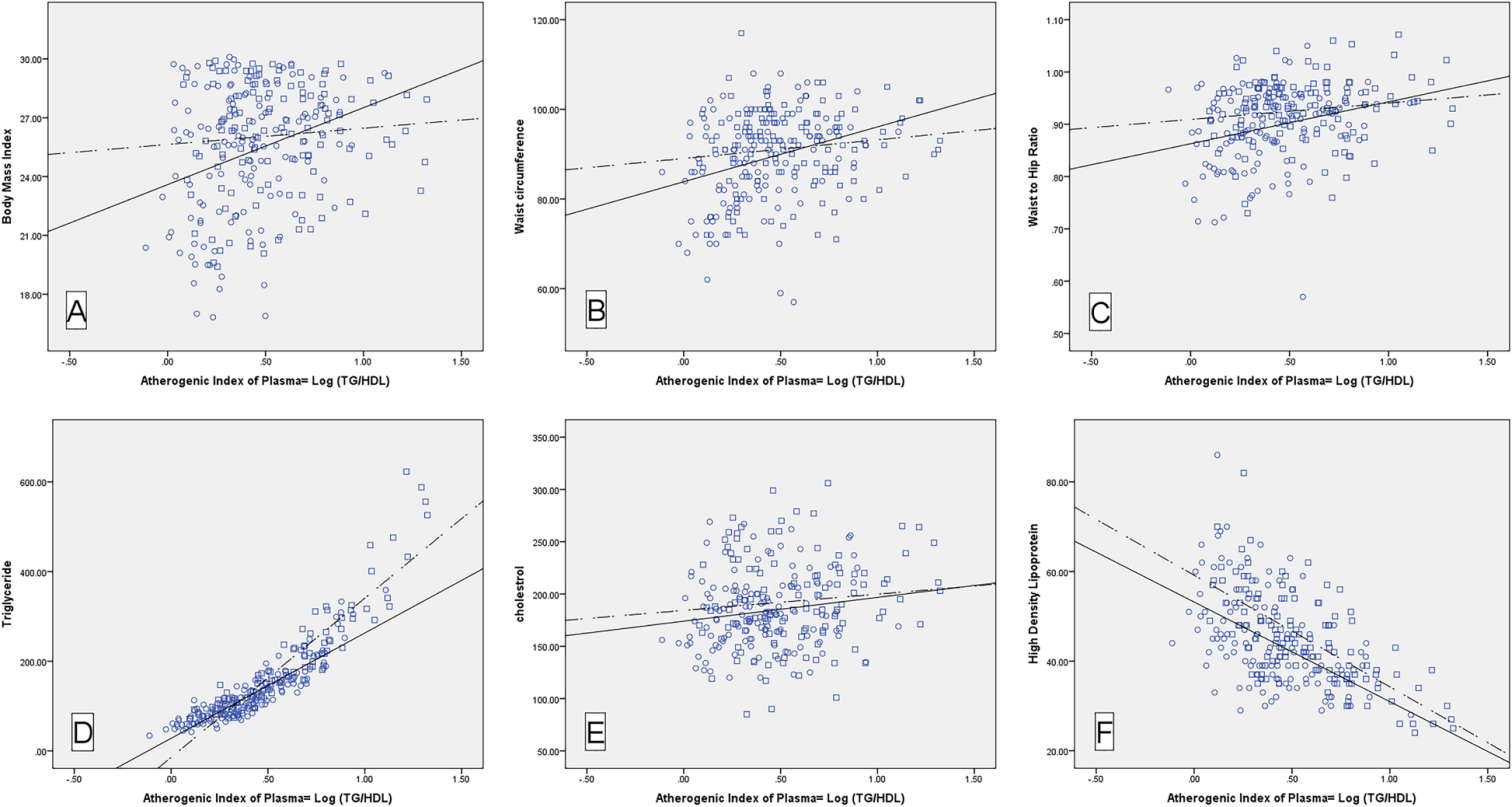
Correlation of AIP with **A**) BMI, **B**) WC, **C**) WHR, **D**) TG, **E**) TC, **F**) HDL-C. * Control: circle; Case: square; Control regression line: solid line; Case regression line: dashed line

Figure 1 indicates the bivariate (Pearson) correlation of AIP with BMI (controls: *R* = 0.281, cases: *R* = 0.089), WC (controls: *R* = 0.289, cases: *R* = 0.134), WHR (controls: *R* = 0.258, cases: *R* = 0.144), TG (controls: *R* = 0.894, cases: *R* = 0.919), TC (controls: *R* = 0.161, cases: *R* = 0.100), and HDL-C (controls: *R* = 0.509, cases: *R* = 0.695). The correlations of AIP with BMI, WC, WHR, TG, and TC are direct relationship, however, the correlation of AIP with HDL-C is inverse relationship.

## Discussion

We sought the association between serum 25(OH) D_3_ levels and atherogenic indices of plasma as novel surrogate markers of cardiometabolic disease risk in patients with DM in a population-based KERCADR cohort study as an Iranian community. We found that cardiometabolic biomarkers significantly decreased for BMI, WC, WHR, FBS, and TG among control males and for WHR, FBS, TG, SBP, and DBP among control females. Gender differences in the cardiometabolic biomarkers have been revealed similar to other studies [20, 21]. In the present study, there were no significant differences in serum 25(OH) D between case and control participants. Unlike, our current findings, in a study, the prevalence of low serum 25(OH) D_3_ level considerably was high in patients with CVD risk factors. These patients presented significantly higher values for cardiometabolic biomarkers [6]. Multiple Regression Model showed that for an individual to maintain metabolic parameters, at least at borderline values, serum 25(OH) D_3_ level should be 37.64 nmol/L [6]. A positive and significant association between AIP and higher HbA1c and lower HDL-C were seen in people with plasma 25(OH) D_3_ less than 25 nmol/L [12]. This finding was parallel to our findings (Data not shown). Moreover, we found a significant increase in AIP for case males and females than control males and females. Although, the other atherogenic indices of plasma increased nearly in cases than controls; there were no significant differences between case and control males and females. Therefore, the results of our study were parallel to the other studies [9–11] that revealed AIP compared to CRI I, CRI II, and AC can be used as a novel surrogate marker; however, the other studies have been shown that AIP is associated with subclinical atherosclerosis and CVD events in participants with DM [8–11]. In a study, the serum 25(OH) D levels were negatively associated with AIP in men but not in women. In addition, vitamin D deficient men had higher AI*P* values than vitamin D sufficient men [16]. Deficient serum 25(OH) D was associated with higher TC, LDL-C, and TG in middle-aged and elderly Chinese individuals. This finding suggested that low 25(OH) D levels was a marker for elevated atherogenic lipoproteins [22]. In another study, the researchers found that AIP was an independent predictor of CAD [23]. One of the most important reasons for the lack of significant differences in these biomarkers may be the lack of significant differences between the cardiometabolic biomarkers that make up these novel biomarkers. The other reasons for the lack of significant differences may be the selection of stringent exclusion criteria as well as the correlation coefficients between AIP and the other biomarkers (Table 6). Interestingly, the levels of AIP, CRI, and AC significantly decreased with improved serum vitamin D status only in control males. Although, the trends of the levels of atherogenic indices were irregular in case and control females with improving vitamin D status; the levels of these indices in case males decreased non-significantly with improving vitamin D status. Vitamin D deficiency in poor glycemic control is likely to develop dyslipidemia as compared to vitamin D insufficient and sufficient groups [7]. A Univariate ANOVA to determine whether the interaction between two independent variables such as three vitamin D statuses and BMI on atherogenic indices of plasma as the dependent variable is significant. We revealed that there was a positive significant association between BMI and the level of AIP with increasing serum vitamin D in control males and females. In other words, the effect of decreasing BMI on significant decrease the level of AIP for control participants with improving serum vitamin D was more effective. An adequate serum vitamin D level might have possible beneficial effects on the level of AIP in normal BMI. Generaly, the mean difference AIP between the case and control females was significant. The researchers observed a negative correlation between 25(OH)D levels and the atherogenic profile in obese patients [18].

The correlation analysis showed a negative linear association between serum 25(OH) D and TG, TC, and LDL-C and a positive linear association between serum 25(OH) D and HDL-C in case and control males but none with females. This current finding was parallel to another study [18].

The results of a meta-analysis showed that lipid and lipoprotein profiles indicate the risk of T2DM; however, the level of AIP might be more closely associated with the risk of T2DM and be used as a predictive indicator in evaluating the risk of T2DM [24]. The findings of another study showed that increasing in AIP was associated with the other CVD risk factors and AIP can be used as a sensitive and regular index of CVD when the other lipid values were within the normal range [25]. The results of our study revealed that the level of AIP among the other atherogenic indices of plasma could a surrogate markers for the incidence of T2DM and CAD in participants with CVD. As the level of AIP was positively associated with CRI I and AC as two novel atherogenic risk markers, they can be used as predictive surrogate markers for CAD/CVD in populations. Although the correlation between CRI II and CRI I was very strong; however, CRI II could not be used as an alternative to AIP for predicting CVD risk in our study. The correlation of AIP with the other cardiometabolic biomarkers was also similar to the other studies [9, 25].

A strong negative correlation between low vitamin D status (serum 25(OH) D < 15 ng/mL) and the three identified biomarkers of atherogenic dyslipidemia: high serum levels of small density LDL-C, TG, and VLDL-C in middle-aged adults without CVD [26]. The findings of our study parallel to the other studies [11, 27] revealed that AIP can be recommended as a novel surrogate marker in the diagnosis of CVD and progression of atherosclerosis in healthy and diabetic participants.

### Strengths and limitation

There are several strengths in the present study. The participants in this nested case-control study were selected from the second phase of a large KERCADR cohort study as an Iranian community. Participants with diabetes and controls were randomly selected and matched by some factors. Exception for glycemic indices and lipid and lipoprotein profiles, case and control groups were matched by the other risk factors such as high blood pressure and BMI equal or greater than 30 and known potential confounders. All possible analyzes were performed between participants with diabetes and controls by gender. One of the most important limitations of the present study was the non-entry of a number of patients with diabetes who had the other risk factors and did not enroll in our investigation. Therefore, with the management of confounding in study design, the cases to controls ratio became 1:1.

## Conclusions

We conclude that there is a reverse significant association between AIP and serum vitamin D among healthy males. Low serum level of vitamin D is associated with atherogenic dyslipidemia. Therefore, improving vitamin D status as an important indicator may alleviate AIP as a surrogate marker for predicting the risk of CVD events in healthy men and women with normal BMI. The effect of decreasing BMI on significant decrease of AIP level for healthy participants with improving serum vitamin D is more effective. The level of AIP among the other atherogenic indices of plasma could a potential predictive biomarker for the incidence of T2DM and CAD in participants with CVD. In general, the level of atherogenic indices of plasma in the controls with normal BMI is lower than the controls with high BMI. This effect is predominant with improving serum vitamin D.

## Data Availability

The data that support the findings of this study are available from the Head of Physiology Research Center, but restrictions apply to the availability of these data, which were used under license for the current study, and so are not publicly available. Data are however available from the authors upon reasonable request and with permission of the Head of Physiology Research Center.
